# We or I, Which?

**Published:** 1879-01

**Authors:** 


### WE OR I, WHICH?

How does it come that an editor when speaking of himself uses the plural pronoun, and when speaking of any other person uses the singular? Is it because the editor usually esteems himself as equally important to two or more of any other people, or is it from custom adopted long ago, and now sanctified by age.

Webster says: "We is often used by individuals, as authors, editors, and the like, in speaking of themselves, in order to avoid the appearance of egotism in the too frequent repetition of the pronoun I." If authors and editors were more sensitive about appearing egotistical than others, there might be an appearance of reason in the quotation.

If egotism and self-conceit were to be abated, or even diminished, by the substitution of *we* for *I*, there is quite a number of the human family who should make the change immediately, and some of them are not editors or authors either.

It is proposed in these pages hereafter, for a time at least, to use I, mine and me, for we, ours and us, and see what the effect will be. Of course if I become too egotistical something must be done about it.

